# From associations to clinical practice: translating inflammatory-nutritional indices into a machine learning-driven model for breast cancer risk stratification with cross-ethnic validation

**DOI:** 10.3389/fimmu.2026.1845883

**Published:** 2026-07-15

**Authors:** Yue Li, Ting Ding, Xiaoyan Zhou, Chao Lu, Yue Zhang, Qian He, Jiangbo Ding

**Affiliations:** 1 Department of Clinical Laboratories, The Second Affiliated Hospital of Xi’an Jiaotong University, Xi’an, China; 2 Jitang College, North China University of Science and Technology, Tangshan, China; 3 Department of Clinical Research, Xianyang Hospital of Yan’an University, Xianyang, China

**Keywords:** breast cancer, inflammation status, machine learning, nutrition, risk stratification

## Abstract

**Objectives:**

To evaluate inflammatory-nutritional indices in relation to breast cancer (BC) risk and mortality and develop a cross-ethnically validated prediction model.

**Methods:**

From National Health and Nutrition Examination Survey (NHANES) 2005–2018, 485 BC patients and 16,838 female controls were included, with mortality follow-up through 2019. Weighted multivariate logistic and Cox regression assessed associations between seven inflammatory indices, two composite indicators, and BC risk/mortality. Multiple machine learning (ML) algorithms, including XGBoost, were used to construct risk models. The model was externally validated (NHANES other periods:1999-2004) and cross-ethnic validated. We prospectively enrolled Chinese treatment-naïve breast cancer patients and matched healthy controls for external validation.

**Results:**

In fully adjusted models, the Advanced Lung Cancer Inflammation Index (ALI) was inversely associated with BC risk and all-cause mortality (highest vs. lowest tertile: odds ratio [OR] 0.64, 95% CI 0.45–0.91; hazard ratio [HR] 0.41, 95% CI 0.18–0.90). Conversely, neutrophil percentage-to-albumin ratio (NPAR), systemic inflammation response index (SIRI), and neutrophil-to-lymphocyte ratio (NLR) showed positive associations. ALI outperformed other indices in predicting mortality. XGBoost identified NPAR as the top predictive feature; the model incorporating inflammatory indices and age achieved an AUC of 0.832 on the test set, and a web-based dynamic nomogram incorporating these factors was developed. External validation yielded AUCs of 0.781 (NHANES) and 0.730 (Chinese cohort).

**Conclusions:**

ALI (protective) and NPAR/SIRI/NLR (detrimental) are robust predictors of BC risk and mortality. The ML model demonstrates good predictive performance, but cross-ethnic validation highlights the need for population-specific calibration, which indicated the potential of ML approaches leveraging inflammatory-nutritional indices to enhance BC risk stratification and inform clinical decision-making.

## 1 Introduction

Breast cancer (BC) remains a critical public health issue, representing the most prevalent cancer among women and a leading cause of cancer-related mortality globally. In recent years, the incidence rates have surged, with an estimated 2.3 million new cases diagnosed in 2020 alone, underscoring the necessity for improved diagnostic and prognostic tools (1). This immense disease burden underscores the urgent need for scalable strategies across the entire cancer care continuum, from primary prevention and early detection to post-diagnosis prognostic stratification. Emerging evidence highlights extratumoral tumor systemic inflammation as a key determinant of survival (2, 3). Inflammatory conditions are frequently present before a malignant tumor occurs, inflammation in the tumor microenvironment has many tumor-promoting effects, including promoting the proliferation and survival of malignant cells, promoting angiogenesis and metastasis, and changing responses to hormones and chemotherapeutic agents (4). Chronic inflammation not only facilitates tumor growth but also impairs the immune system’s ability to detect and eliminate malignant cells, fostering an environment conducive to cancer progression (4–6). Hematologic indices, such as the neutrophil-to-lymphocyte ratio (NLR), platelet-to-lymphocyte ratio (PLR), monocyte-to-lymphocyte ratio (MLR) and neutrophil-to-platelet ratio (NPR) can serve as accessible surrogates of this systemic state.

The biomarker landscape has since evolved to include more complex, multi-dimensional indices that aim to capture the interplay between inflammation and other host factors. The Systemic Immune-Inflammation Index (SII) and Systemic Inflammation Response Index (SIRI), for instance, integrate neutrophils, platelets, and lymphocytes to offer a more comprehensive reflection of the systemic inflammatory response. The Aggregate Index of Systemic Inflammation (AISI) further broadens this spectrum. Critically, the host’s nutritional status is increasingly recognized as a key modulator of inflammation and cancer outcomes. Indices like the Advanced Lung Cancer Inflammation Index (ALI) and the novel Neutrophil Percentage-to-Albumin Ratio (NPAR) explicitly combine inflammatory markers with albumin, a key nutritional protein, providing an integrated assessment of inflammatory-nutritional balance.

Preliminary studies have associated these markers with outcomes in lung, colorectal, and pancreatic cancers, yet their utility in breast cancer remains underexplored (7–9). Findings have indicated that both SIRI and ALI may serve as significant predictors of survival among adult cancer survivors, reinforcing the importance of monitoring inflammatory status in the management of BC (10, 11). In recent years, AISI receives widespread attention as a comprehensive inflammatory indicator reflecting the inflammatory state, and its close association with cardiovascular disease and pan-cancer have been found (9, 12), but its association with breast cancer has not been specifically studied. Existing literature is fragmented, with studies typically examining one or two indices in isolation and therefore fail to determine their relative predictive strength and address their potential utility in risk assessment. Furthermore, the high degree of formulaic overlap among certain indices (e.g., SII, SIRI, AISI) raises valid concerns about collinearity and redundant information, an issue that previous studies have seldom addressed methodologically.

To bridge these gaps, we leveraged the nationally representative National Health and Nutrition Examination Survey (NHANES) to conduct a comprehensive analysis of a broad panel of nine inflammatory and inflammatory-nutritional indices—SIRI, SII, ALI, AISI, NLR, PLR, MLR, NPR and NPAR. Our study has three primary aims: First, to rigorously evaluate and compare the associations of these indices with breast cancer risk in a cross-sectional design, which is methodologically appropriate for assessing diagnostic or risk factors. Second, to perform the comparison of these indices within a unified BC cohort to identify optimal predictors and determine these associations with BC mortality by weighted Cox regression analysis and Kaplan-Meier survival method. Finally, we identified the most potent and non-redundant biomarkers through machine learning (ML) techniques, which are robust to multicollinearity, for the development of a robust diagnostic prediction model, and externally validated this model to ensure its generalizability and potential clinical utility for early detection and risk stratification. These findings could ultimately inform future targeted interventions that mitigate the inflammatory processes associated with BC and improve patient outcomes.

## 2 Methods

### 2.1 Study population and data collection

**Figure 1:**
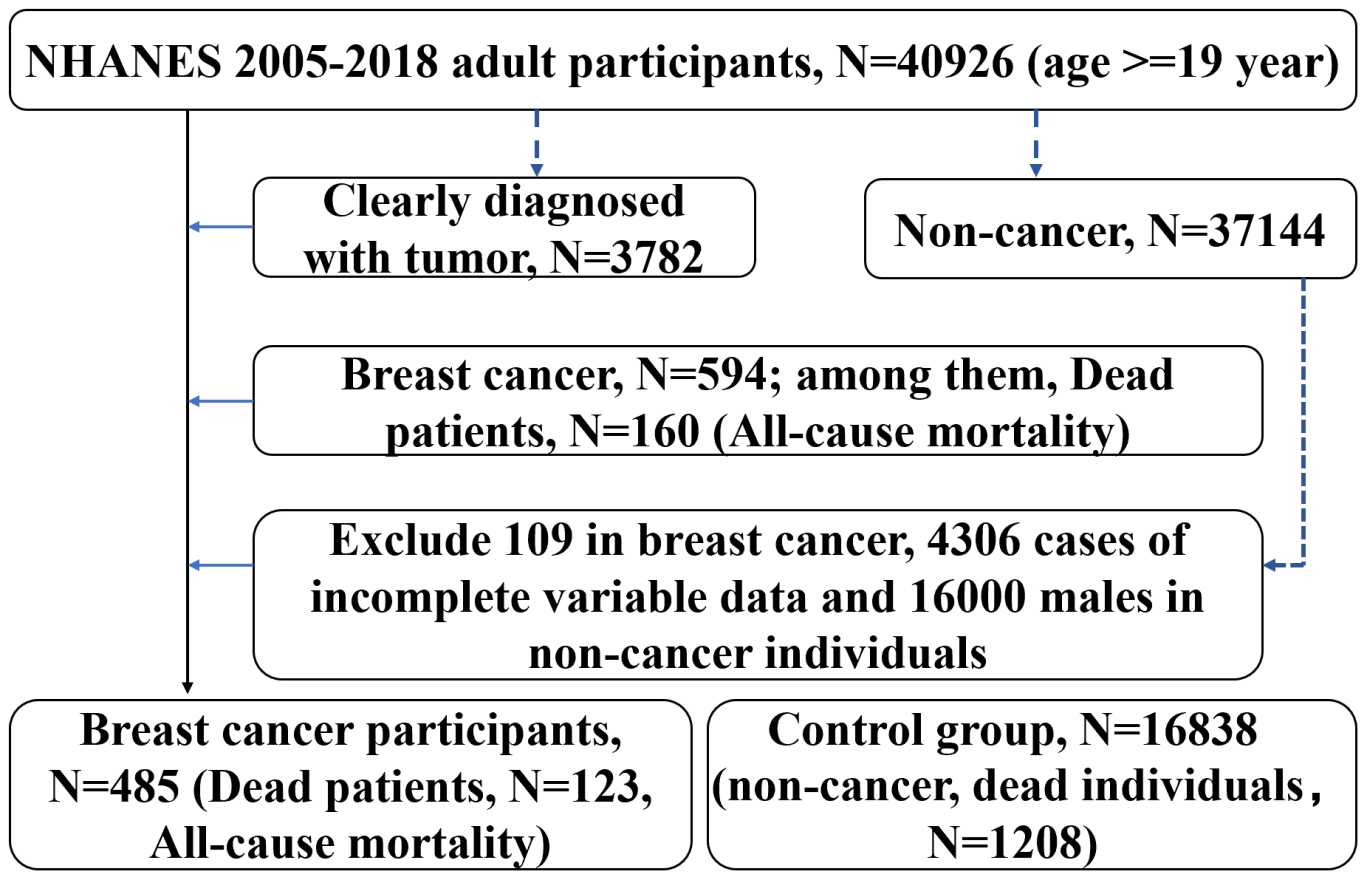
Selection process for study cohorts.

Public data from NHANES for the years 2005 to 2018 were collected for the current study (https://wwwn.cdc.gov/nchs/nhanes/default.aspx, accessed on 10 November 2024). The Publicly open-data from the NHANES database including the physical, nutritional and health status of adults and children in the United States, has been used and analyzed by many studies. The Research Ethics Review Boards of the National Center for Health Statistics and the Centers for Disease Control and Prevention approved the survey plan. Written informed consent was provided by all participants or proxies, who were selected through a complex multistage probability sampling design. Standard demographic data were collected. Breast cancer was self-reported by individuals, and was acquired by NHANES question mcq220, mcq230a, mcq240e: “Have you ever been told by a doctor or other health professional that you had cancer or a malignancy of any kind?”, “1st cancer - what kind was it?”, “How old were you when breast cancer was first diagnosed?”. Inclusion criteria for this study were as follows: 1) individuals diagnosed with breast cancer; 2) age above 19 years. Exclusion criteria were as follows: 1) lack of data records on survival status and follow-up time; 2) missing data on key covariates including blood routine, albumin test data, and BMI, and further calculated values (SII, SIRI, ALI and AISI). The exclusion criteria for the control group were the same as above. Also, through NHANES question mcq230a, we only analyzed the participants whose first malignant tumor was breast cancer, without considering secondary tumors (breast cancer). Finally, a total of 485 individuals over the age of 19 with breast cancer were encompassed in the current study. **Figure 1** illustrated the detailed screening steps.

### 2.2 Definition of inflammatory markers

The systemic inflammatory response is associated with survival in patients with a variety of cancers. This inflammatory response is measured in the peripheral blood, and can be monitored using two categories of indices: concentration of specific serum proteins (albumin, C-reactive protein) and differential blood cell count (neutrophils, lymphocytes and platelets) (13). SII, SIRI and AISI, two novel composite indices integrating three independent white blood cell subsets and platelets, and ALI integrating inflammatory and nutritional biomarkers, which all have shown promising potentials in predicting outcomes in various diseases (14). SIRI was calculated by the formula: (neutrophil count × monocyte count)/lymphocyte count, while that of SII was: (platelet count × neutrophil count)/lymphocyte count (15), the AISI formula was calculated as (neutrophil count × monocyte count × platelet count)/lymphocyte count, and ALI with the specific formula being BMI (kg/m2) * albumin level (g/dL)/NLR. The NPAR index was calculated as neutrophil percentage(%)×100/albumin level (g/dL). NLR was calculated as neutrophil counts divided by lymphocyte counts. In addition, we also calculated MLR (monocyte counts/lymphocyte counts), PLR (platelets/lymphocyte counts), NPR (neutrophils/platelets).

### 2.3 Covariates definitions and collections

The demographic characteristics of participants were extracted (age, sex, race, education level, marital status and poverty income ratio (PIR)), based on standardized questionnaires. And data on body mass index (BMI, kg/m^2^) were from the body measures (weight and height) collected in the mobile examination center. Additionally, Albumin, neutrophil counts, lymphocytes, monocytes and platelets, were collected from laboratory measurements. Smoking (SMQ020: Smoked at least 100 cigarettes in life) and alcohol (ALQ101/ALQ142) consumption were dichotomized into yes or no categories. Diabetes was identified based on a medical history of the participants’ records. The missing data for these covariates were excluded from the current study.

### 2.4 Survival status ascertainment

Survival outcomes were determined by correlation of NHANES public-use linked mortality file with National Death Index records by a probabilistic matching method (https://www.cdc.gov/nchs/data-linkage/mortality-public.htm, accessed on 18 November 2024), updated on December 31, 2019. The primary cause of death was recorded using the International Statistical Classification of Diseases and Related Health Problems, Tenth Revision (ICD-10), and primary outcomes of the data included all-cause, cancer-specific, and noncancer mortality. All-cause mortality was defined as death from all causes; cardiovascular mortality was defined as death from heart disease (ICD-10 codes 054–068); cancer-specific mortality as ICD-10 codes 019-043; and deaths from other causes as noncancer mortality. Referring to some research (10, 16), the number of months from the interview date to the date of death or, for individuals who did not suffer an event, through December 31, 2019, was defined as the follow-up period survival (FPS). Meanwhile, based on the interview including “How old were you when cancer was first diagnosed?” and “How old were you when breast cancer was first diagnosed?”, the overall survival (OS) was defined from the first diagnosed date of cancer to the date of death or December 31, 2019.

### 2.5 Statistical analysis for breast cancer risk and mortality

The significance level for all tests was set at *P* < 0.05. All analyses were conducted using R version 4.3.3, and software graphpad prism 9, SPSS 21.0 and EmpowerStats 4.2 (www.empowerstats.com, accessed on June 2025).

#### 2.5.1 Association between inflammatory markers and breast cancer risk

Continuous variables were compared using the Kruskal-Wallis rank sum test, while categorical variables were compared using the Chi-square test or Fisher’s exact test as appropriate. To analyze the association between inflammatory markers and breast cancer risk, these markers were categorized into tertiles (T1: low, T2: medium, T3: high). Weighted multivariate logistic regression models were applied to calculate Odds Ratios (ORs) and 95% Confidence Intervals (CIs) across these categories. Three models were constructed: Model 1 (unadjusted); Model 2 (adjusted for age and race); Model 3 (fully adjusted for age, race, education, marital status, PIR, BMI, and diabetes).

The potential nonlinear relationships were explored using the Generalized Additive Model (GAM) with penalized splines. If a nonlinear relationship was identified, a two-piecewise linear regression model was fitted using a recursive algorithm to determine the inflection point. Stratified analyses were also performed across subgroups defined by age, race, education, marital status, PIR, BMI, and diabetes status. We used Receiver operating characteristic curve (ROC) curves to evaluate the predictive performance of these inflammatory markers for breast cancer risk.

#### 2.5.2 Association between inflammatory markers and mortality among breast cancer patients

Among breast cancer patients, the relationship between inflammatory markers (categorized into tertiles T1-T3) and all-cause mortality was evaluated using weighted multivariate Cox proportional hazards models, with adjustments consistent with the logistic regression models. Survival probability differences were assessed using Kaplan-Meier curves with log-rank tests. Restricted cubic spline (RCS) curve was used to identify potential nonlinear relationships. The predictive performance of inflammatory markers for mortality was evaluated using time-dependent ROC curves.

### 2.6 Multiple machine learning algorithm models for breast cancer patient

We employed multiple machine learning algorithms for binary classification to distinguish breast cancer risk (defined as the presence of a self-reported physician diagnosis of breast cancer in NHANES participants), including logistic regression (LR), gradient boosting machine (GBM), extreme gradient boosting (XGBoost), random forest (RF), decision tree (DT), support vector machine (SVM), naive Bayes (NB), and K-nearest neighbors (KNN). Based on comparative performance evaluation and overfitting assessment, XGBoost was selected for final model development due to its superior and stable predictive accuracy, leveraging the efficient Gradient Boosting Decision Tree algorithm for supervised learning tasks.

For model development, we constructed an exploration cohort comprising all 485 breast cancer cases and 561 randomly selected female controls from the non-cancer population. This specific sampling strategy was necessary because the original control pool (n = 16,838) substantially outnumbered the cases, creating a severe class imbalance that could bias ML algorithms toward the majority (control) class and compromise their ability to discriminate breast cancer cases. By selecting a control subset of comparable size (561 vs. 485) to the case group, we achieved a more balanced dataset for training and evaluating the prediction models. The total exploration cohort therefore consisted of 1,046 participants, which was then randomly partitioned into a training set (85%) and a testing set (15%). We evaluated model performance through 5−fold cross-validation on the training set, with grid search employed for hyperparameter optimization. We compared machine learning classifiers, including XGBoost, logistic regression, random forest, etc., evaluated their performance through 5-fold cross validation, and used grid search for hyperparameter optimization. The training set used 5-fold cross-validation and were separated into five folds, using five of the folds as the training set to train the model, and the remaining one-fold as the internal validation set to score the model, and repeating the above process five times.

The prediction models were constructed using the Beckman Coulter DxAI platform (https://www.xsmartanalysis.com/beckman/login/, accessed on 1 July 2025) and python (xgboost,2.0.1) (17). Shapley additive explanation (SHAP) analysis was utilized to indicate the significance and contributions of features within the ML models. Area under the ROC curve (AUC), accuracy, sensitivity, specificity, positive predictive value and negative predictive value were calculated to determine the model’s performance (17). To obtain a less biased estimate of the model’s predictive performance while accounting for the entire modeling process (including hyperparameter tuning), we additionally performed nested cross-validation on the dataset (18). This nested strategy comprised an outer loop of 5−fold cross−validation for performance evaluation and an inner loop of 5−fold cross−validation for hyperparameter selection within each training fold. The mean AUC across the outer folds was calculated as the final performance estimate to reduce potential optimism.

### 2.7 The independent validation of breast cancer risk prediction ML models

Firstly, the study used all NHANES data (2005-2018, **Figure 1**) mentioned earlier as the validation set to analyze the prediction ability of ML risk model (XGBoost model). Secondly, we utilized other periods in the NHANES database (1999-2000, 2001-2002, 2003-2004 and 2017-March 2020 Pre-Pandemic) as an independent validation set to analyze the prediction ability of ML risk model. The methods for collecting and organizing data were the same as above.

### 2.8 The cross-ethnic external validation of breast cancer risk prediction ML models

Furthermore, we conducted an external validation using an independent cohort recruited from The Second Affiliated Hospital of Xi’an Jiaotong University. The study was approved by the ethics committee of Xi’an Jiaotong University Second Affiliated Hospital (2025-212). This cross-sectional study did not involve any personal, private information about the patient and only an analysis of available test data was performed. The committee waived the need for individual informed consent.

The validation cohort comprised two distinct groups (breast cancer: N = 98; control: N = 103). The cases group included treatment-naïve patients with newly diagnosed breast cancer, admitted between September 2025 and January 2026. Participants were enrolled based on initial clinical presentations such as breast lumps/masses or abnormal findings in physical examinations requiring further investigation. All breast cancer diagnoses were pathologically confirmed. The control group consisted of individuals from the same period undergoing routine health check-ups. These controls were determined to be in good health, with normal physiological functions (e.g., cardiac and pulmonary) and laboratory parameters, and with no history of any malignancy.

## 3 Results

### 3.1 Baseline characteristics

**Figure 2:**
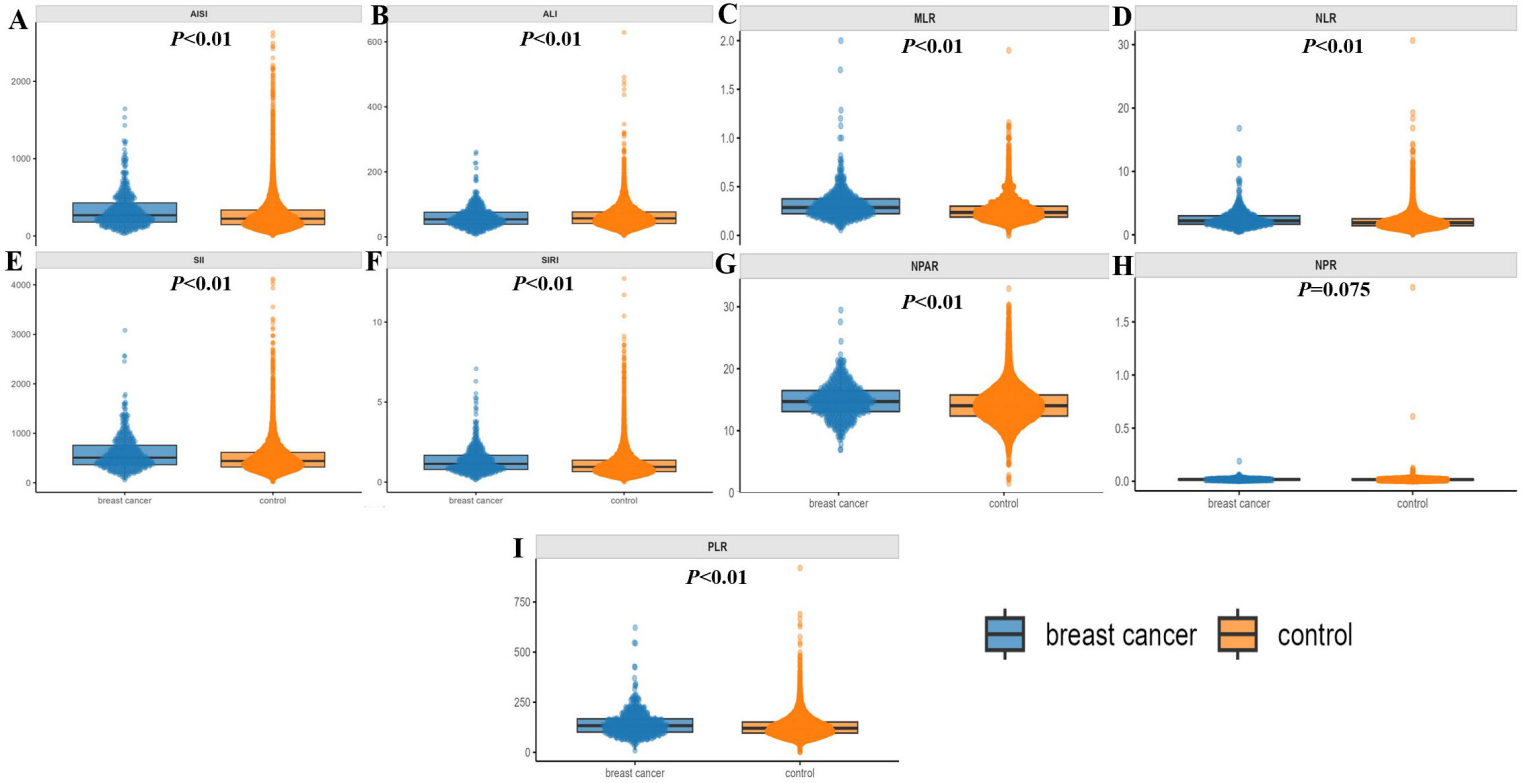
Distribution of inflammatory markers between breast cancer and control group. **(A–I)** Distribution of inflammatory markers between breast cancer and control group, including AISI **(A)**, ALI **(B)**, MLR **(C)**, NLR **(D)**, SII **(E)**, SIRI **(F)**, NPAR **(G)**, NPR **(H)**, PLR **(I)**.

A total of 40926 participants [age = 48.83 (18.48), (median (SD)] were included, and participant characteristics were shown by cancer/BC status in **Supplementary Table 1** and **Table 1**. Compared with non-cancer group, we found that participants of older age (≥ 60 year), non-Hispanic white, college or above, PIR > 3.50, widowed/divorced/separated, diabetes were more likely to develop cancer (*p* < 0.05). Through inclusion and exclusion criteria, 485 breast cancer patients and 16838 female control participants were enrolled, and the results showed that BC group were older, had a more proportion of non-Hispanic white, widowed/divorced/separated individuals and diabetes patients, a higher PIR (*p* < 0.05, **Table 1**). There was no significant difference in variables such as BMI, education, smoking and alcohol status. As shown in **Table 1** and **Figure 2**, There was a significant difference in ALI levels between BC patients [mean (95% CI of mean): 60.72 (57.63, 63.82)] and control group (*p* < 0.001), as well as the higher SIRI, SII, AISI, NPAR, MLR, NLR, PLR levels in BC compared with control (*p* < 0.001), without significant difference in NPR between breast cancer and control group (*p* = 0.075).

**Table 1:** Baseline characteristics of breast cancer participants and control group.

Characteristics	Control (n = 16838)	Breast cancer (n = 485)	P-value
Age (mean ± SD)	46.97 ± 17.81	66.97 ± 11.56	<0.001
Age_level (%)			<0.001
[19,40)	6525 (38.75%)	12 (2.47%)	
[40,60)	5532 (32.85%)	105 (21.65%)	
≥ 60	4781 (28.39%)	368 (75.88%)	
Race, (%)			<0.001
Mexican American	2881 (17.11%)	51 (10.52%)	
Other Hispanic	1813 (10.77%)	37 (7.63%)	
Non-Hispanic White	6491 (38.55%)	287 (59.18%)	
Non-Hispanic Black	3701 (21.98%)	77 (15.88%)	
Other Race	1952 (11.59%)	33 (6.80%)	
BMI,g/m2	29.54 ± 7.68	29.42 ± 6.98	0.740
Education, n(%)			0.370
Less than high school	3962 (24.22%)	104 (21.44%)	
High school or equivalent	3563 (21.78%)	110 (22.68%)	
College or above	8832 (54.00%)	271 (55.88%)	
PIR	2.41 ± 1.62	2.74 ± 1.62	<0.001
PIR, n(%)			<0.001
< 1.30	5161 (33.91%)	108 (24.71%)	
1.30–3.50	5701 (37.46%)	176 (40.27%)	
> 3.50	4356 (28.62%)	153 (35.01%)	
Marital status, n(%)			<0.001
Married/Living with partner	9124 (55.35%)	243 (50.10%)	
Widowed/Divorced/Separated	4304 (26.11%)	218 (44.95%)	
Never married	3056 (18.54%)	24 (4.95%)	
Smoking, n (%)			0.068
Yes	10912 (65.97%)	300 (61.98%)	
No	5629 (34.03%)	184 (38.02%)	
Alcohol, n (%)			0.327
Yes	8566 (51.56%)	261 (53.81%)	
No	8049 (48.44%)	224 (46.19%)	
Diabetes, n (%)			<0.001
No	14614 (88.69%)	370 (79.06%)	
Yes	1864 (11.31%)	98 (20.94%)	
SIRI	1.15 ± 0.82	1.41 ± 1.01	<0.001
SII	558.83 ± 341.35	624.01 ± 442.35	<0.001
ALI	70.59 ± 41.45	60.72 ± 34.68	<0.001
AISI	307.90 ± 253.13	348.37 ± 289.83	<0.001
NPAR	14.21 ± 2.95	14.81 ± 2.81	<0.001
MLR	0.26 ± 0.11	0.32 ± 0.18	<0.001
NLR	2.11 ± 1.11	2.55 ± 1.57	<0.001
PLR	128.71 ± 49.53	143.70 ± 66.19	<0.001
NPR	0.02 ± 0.02	0.02 ± 0.01	0.075

Results in table were represented as Mean ± SD or N(%). Kruskal Wallis Rank Test was performed for continuous variables, and Fisher Exact was performed for categorical variables with Expects<10.

### 3.2 Association of inflammatory markers with the odds of breast cancer

Variance inflation factors (VIFs) among all inflammatory markers and covariates were confirmed to be below 5, indicating no significant multicollinearity. The associations between inflammatory markers and breast cancer risk, categorized into tertiles (T1: low, T2: medium, T3: high), were summarized in **Tables 2**, **3**. In the fully adjusted model (Model 3, adjusted for age, race, education, marital status, PIR, BMI and diabetes), several markers showed significant associations with breast cancer risk.

**Table 2:** Association of the two composite indicators of inflammation-nutritional status (ALI and NPAR) and breast cancer risk among US participants, NHANES, 2005 to 2018.

Variables	Exposure		Breast cancer OR (95%CI, p-value)		
	Control (n = 16838)	BC (n = 485)	Model 1	Model 2	Model 3
ALI					
Per ln-unit			0.99 (0.98, 0.99) ***	0.99 (0.98, 0.99) ***	0.99 (0.98, 0.99), **
Low (T1)	5554(32.98)	220(45.36)	Reference	Reference	Reference
Medium (T2)	5627(33.42)	147(30.31)	0.63 (0.46, 0.87) **	0.67 (0.48, 0.93), 0.01	0.75 (0.53, 1.06), 0.11
High (T3)	5657(33.60)	118(24.33)	0.55(0.40, 0.75) ***	0.59(0.43, 0.80) ***	0.64(0.45, 0.91), 0.01
P for trend		<0.001	<0.001	<0.001	0.014
NPAR					
Per ln-unit			1.07 (1.03, 1.11) ***	1.08(1.04, 1.13) ***	1.08 (1.03, 1.13) **
T1	5655(25.23)	117(24.12)	Reference	Reference	Reference
T2	5612(24.98)	159(32.78)	1.25 (0.93, 1.70),0.15	1.23 (0.90, 1.68), 0.19	1.20 (0.86, 1.68), 0.27
T3	5571(25.11)	209(43.09)	1.77 (1.29, 2.42) ***	1.69(1.24, 2.29), 0.001	1.62 (1.15, 2.29) **
P for trend		<0.001	<0.001	<0.001	0.006

Model 1 was unadjusted for none. Model 2 was adjusted for age, race, and Model 3 was adjusted for age, race, education, marital status, PIR, BMI and diabetes. ***P < 0.001; **P < 0.01.

**Table 3:** Association of the six inflammatory markers (SIRI, SII, AISI, MLR, NLR and PLR) and breast cancer risk among US participants, NHANES, 2005 to 2018.

Variables	Exposure		Breast cancer OR (95%CI, p-value)		
	Control (n = 16838)	BC (n = 485)	Model 1	Model 2	Model 3
SIRI					
Per l-unit			1.33 (1.23, 1.44) ***	1.25 (1.14, 1.38) ***	1.22 (1.10, 1.37) ***
T1	5592(33.21)	105 (21.65)	Reference	Reference	Reference
T2	5694(33.82)	155 (31.96)	1.31 (0.94, 1.85),0.11	1.13(0.80, 1.61), 0.49	1.26 (0.85, 1.84), 0.24
T3	5552(32.97)	225 (46.39)	2.24 (1.64, 3.06)***	1.76 (1.29, 2.39) ***	1.83 (1.30, 2.57)***
P for trend		<0.001	<0.001	<0.001	<0.001
SII					
l-unit			1.00 (1.00, 1.00) ***	1.00 (1.00, 1.00) ***	0.99(0.98, 0.99) **
T1	5592(33.21)	148 (30.52)	Reference	Reference	Reference
T2	5694(33.82)	151 (31.13)	0.92(0.67, 1.26), 0.60	0.89(0.64, 1.22),0.46	0.83 (0.59, 1.15), 0.26
T3	5552(32.97)	186 (38.55)	1.33(0.98, 1.80), 0.07	1.40(1.04, 1.89),0.03	1.36 (0.97, 1.91), 0.07
P for trend		0.059	0.06	0.03	0.07
AISI					
1-unit			1.00 (1.00, 1.00) ***	1.00 (1.00, 1.0)**	0.99 (0.98, 0.99)**
T1	5645(33.53)	129(26.60)	Reference	Reference	Reference
T2	5608(33.31)	166(34.23)	1.21(0.89. 1.67), 0.23	1.15(0.84, 1.59), 0.38	1.16(0.82, 1.65), 0.40
T3	5585(33.17)	190(39.18)	1.49(1.12, 1.99),0.008	1.40(1.05, 1.87), 0.03	1.44(1.05, 1.98), 0.03
P for trend		0.002	0.007	0.022	0.023
NLR					
1-unit			1.23(1.15, 1.32) ***	1.16(1.08, 1.25) ***	1.19(1.10, 1.29) ***
T1	5665(33.64)	107(22.06)	Reference	Reference	Reference
T2	5612(33.33)	151(31.13)	1.19(0.87, 1.65),0.28	1.16(0.84, 1.61), 0.36	1.18(0.83, 1.66), 0.36
T3	5561(33.03)	227(46.80)	2.13(1.50, 3.01)***	1.88(1.33, 2.65)***	1.76(1.19, 2.60) **
P for trend		<0.001	<0.001	<0.001	0.004
MLR					
1-unit			10.75 (8.02, 91.17) ***	10.75 (5.52, 20.95) ***	10.51(4.96,22.28) ***
T1	4952(29.41)	76(15.67)	Reference	Reference	Reference
T2	6352(37.72)	150(30.93)	1.83(1.28, 2.65), 0.001	1.45(1.00, 2.10),0.05	1.42(0.94, 2.13), 0.09
T3	5534(32.87)	259(53.40)	3.93(2.79, 5.53)***	2.31(1.63, 3.29)***	2.28(1.53, 3.40)***
P for trend		<0.001	<0.001	<0.001	<0.001
PLR					
1-unit			1.00(1.00, 1.00) ***	1.00(1.00, 1.00) ***	1.00(1.00, 1.00) ***
T1	5632(33.45)	139(28.66)	Reference	Reference	Reference
T2	5643(33.51)	133(27.42)	0.87(0.61. 1.22), 0.42	0.83(0.59, 1.17), 0.29	0.88(0.60, 1.28), 0.49
T3	5563(33.04)	213(43.92)	1.70(1.25, 2.32)**	1.47(1.07, 2.02), 0.01	1.46(0.99, 2.15), 0.05
P for trend		<0.001	0.001	0.01	0.03

Model 1 was unadjusted for none. Model 2 was adjusted for age, race, and Model 3 was adjusted for age, race, education, marital status, PIR, BMI and diabetes. ***p < 0.001; **P < 0.01.

Specifically, elevated levels of the ALI were associated with a reduced risk of breast cancer. Participants in the highest tertile (T3) of ALI had a 36% lower prevalence of breast cancer compared to those in the lowest tertile (T1) (OR, 0.64, 95% CI 0.45–0.91, *P* for trend = 0.014; **Table 2**). Conversely, higher levels of the NPAR, SIRI, AISI, NLR, and MLR were significantly associated with an increased risk of breast cancer. For SIRI, the highest tertile (T3) was associated with an 83% increased risk (OR 1.83, 95% CI 1.30–2.57, *P* for trend < 0.001; **Table 3**), in the fully adjusted model (Model 3). Similarly, the highest tertile of NLR was associated with a 76% increased risk (OR: 1.76; 95% CI: 1.19–2.60; *P* for trend = 0.004), and the highest tertile of MLR was associated with a 128% increased risk (OR: 2.28; 95% CI: 1.53–3.40; *P* for trend < 0.001). Also, the correlation between AISI and breast cancer risk was weak in Model 2 and 3. Other markers, including SII and PLR, also showed positive but less consistent associations with breast cancer risk across models. For instance, while the highest tertile of SII showed a trend toward increased risk (OR: 1.36; 95% CI: 0.97–1.91), the trend was not statistically significant in Model 3 (*P* for trend = 0.07).

**Figure 3:**
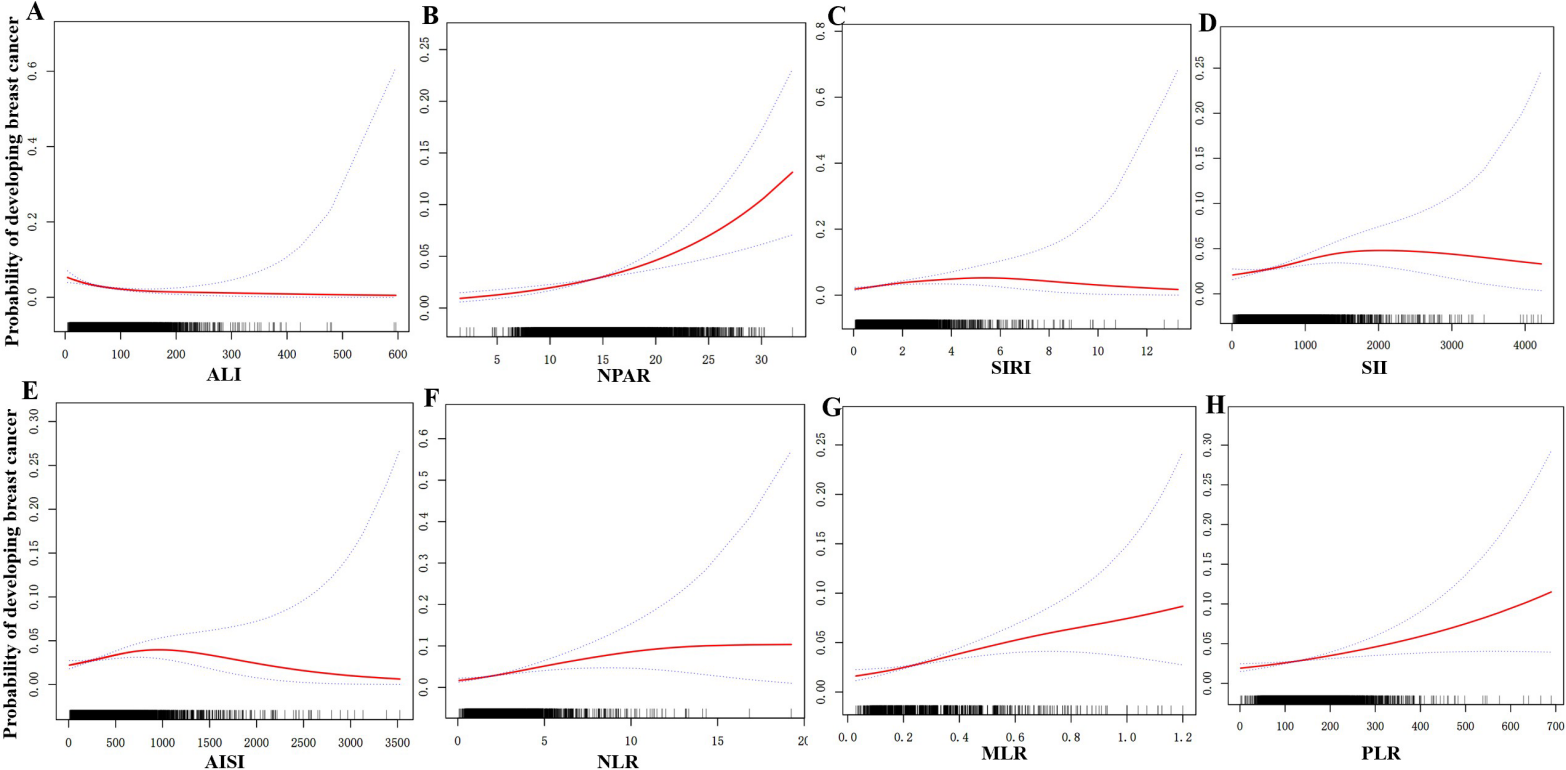
Exploration of the association between levels of inflammatory indicators and BC risk by generalized additive models. Generalized additive models depict the nonlinear association between inflammatory indices and BC risk. The Y-axis represents the predicted BC risk from the GAM model, and the X-axis represents the level of inflammatory indicators: **(A–H)** The association of ALI, NPAR, SIRI, SII, AISI, NLR,MLR and PLR levels with Odds ratio of BC. The blue lines embody the estimated ORs of BC risk with 95% CIs. All covariates (age, race, education, marital status, PIR, BMI and diabetes), consistent with Model 3, were adjusted in this model.

By smoothed curve fitting, a nonlinear relationship was observed between the inflammatory markers levels and BC risk in the adjusted model (**Figure 3**). Fitting by weighted regression adjusted model and two-piecewise regression model, the results indicated there was threshold effects between SIRI, SII or AISI and BC risk and the corresponding inflection points, e.g., 1.461 for SIRI; Below this threshold, each 1-unit SIRI increase elevated BC risk by 83% (95%CI: 1.35-2.49; *p* < 0.001; **Supplementary Table 2**). As shown in **Supplementary Figure 1**, the results of the subgroup analyses by age, race, education and marital status showed that in individuals representing Non-Hispanic White/Black, College or above, PIR 1.30–3.50, normal weight/obese, or Widowed/Divorced/Separated, or those without diabetes, SII, SIRI and AISI displayed a more pronounced positive correlation with breast cancer, as well as a more pronounced negative correlation between ALI and BC.

### 3.3 Association of the inflammation markers with breast cancer all-cause mortality

**Figure 4:**
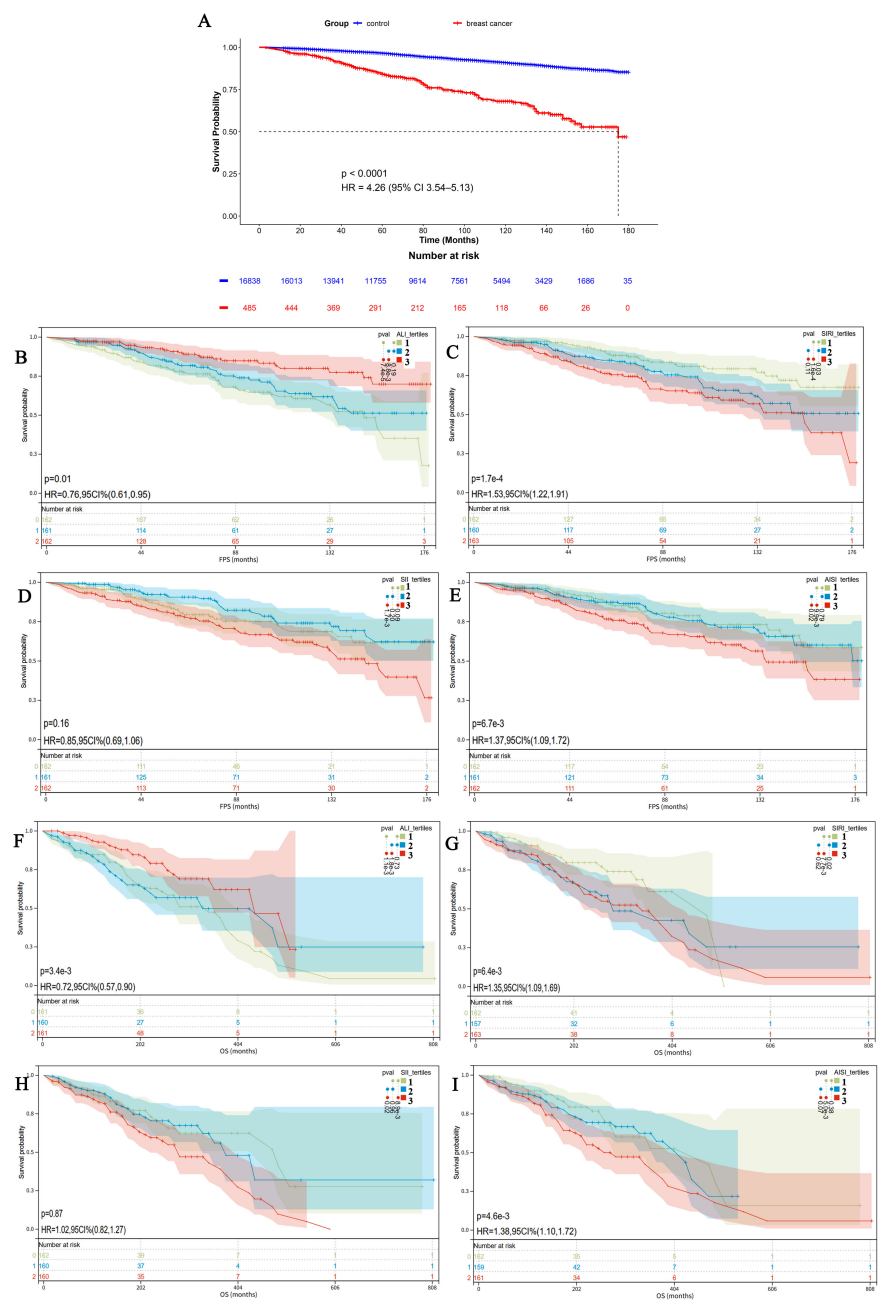
Association of the inflammation markers with breast cancer all-cause mortality based on the Kaplan-Meier survival analysis. **(A)** Survival analysis between breast cancer group and female non-cancer group. **(B–I)** Kaplan-Meier survival curves for all-cause mortality follow-up period survival (FPS) stratified by inflammation markers tertiles. HR, hazard ratio.

The Kaplan-Meier survival analysis showed a significantly lower survival rate in the breast cancer group compared to the control during the follow-up period (*P* < 0.01, **Figure 4A**). In the follow-up data including 485 BC patients, 123 patients were dead. Lower tertiles of ALI (**Figure 4B**), and the highest tertile (T3) of NPAR, SIRI, AISI, NLR, MLR were associated with unfavorable outcome, reduced FPS (**Figures 4C–I**) and OS (**Supplementary Figure 2**) survival period, highlighting the closely association between decreased ALI level, elevated NPAR/SIRI/AISI/NLR/MLR levels and increased all-cause mortality among adult BC patients (for example, T3 vs. T1, *P* < 0.01).

**Figure 5:**
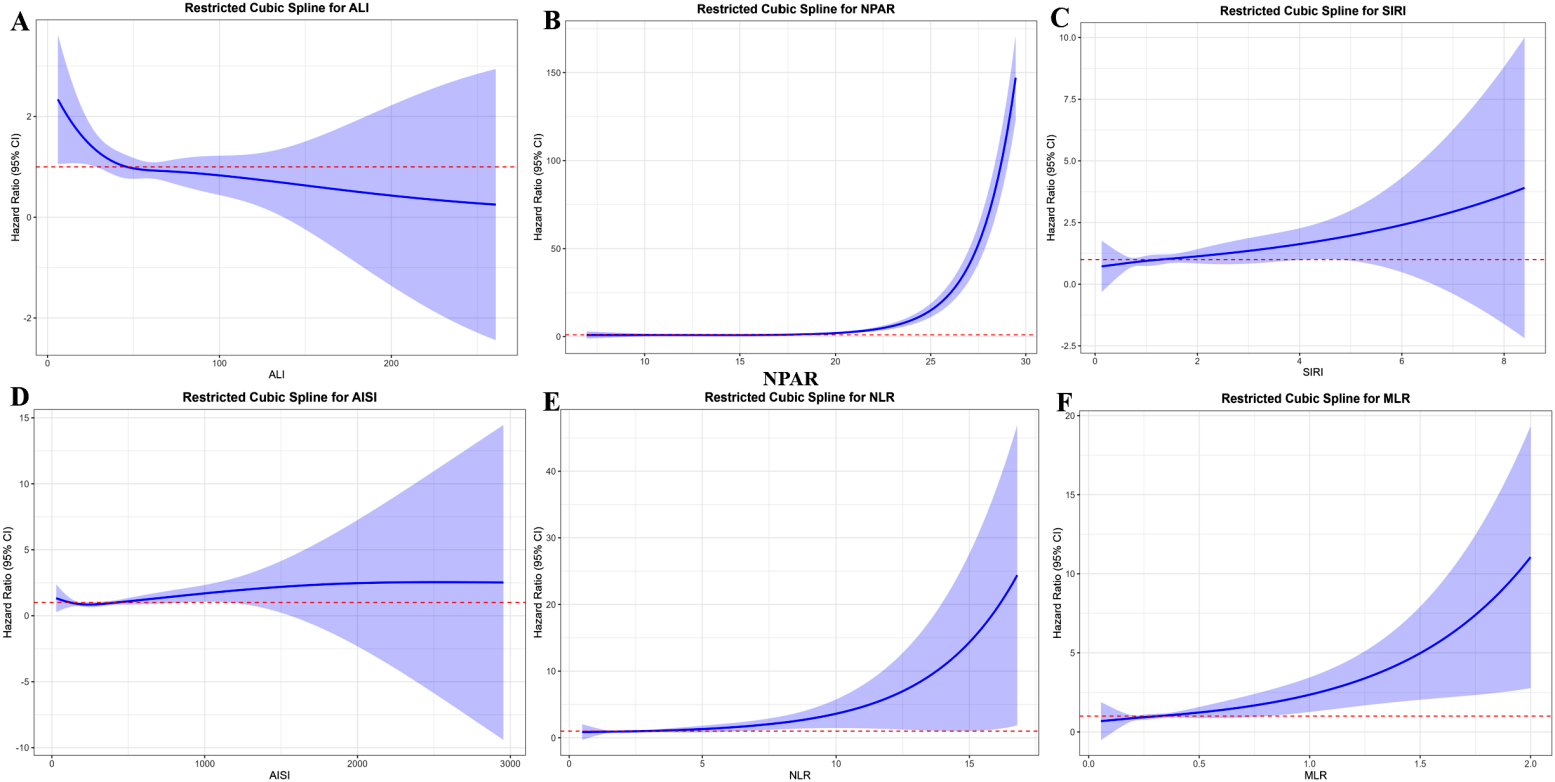
Restricted cubic spline curve for the association of inflammation markers with breast cancer mortality. **(A–F)** The association of ALI, NPAR, SIRI, AISI, NLR,MLR levels with Hazard ratio of BC. The purple lines embody the estimated HRs of BC mortality with 95% CIs. All covariates (age, race, education, marital status, PIR, BMI and diabetes), consistent with Model 3, were adjusted in this model.

We further evaluated the prognostic value of inflammatory markers for all-cause mortality in breast cancer patients using weighted Cox regression models (**Table 4**). In the fully adjusted model (Model 3), ALI demonstrated significant associations with BC mortality risk. Based on the baseline characteristics of ALI level (70.59 ± 41.45, in control), each 10-unit increase in ALI was linked to a 13% reduction in all-cause mortality risk (HR: 0.87, 95% CI: 0.79-0.97, *P* = 0.012). Accordingly, patients in the highest ALI tertile (T3) showed a 59% lower risk of death compared to those in the lowest tertile (T1) (HR: 0.41; 95% CI: 0.18-0.90; *P* for trend = 0.02). This suggested that ALI was not only an indicator negatively correlated with BC risk but also serves as a predictor of prognosis. For other markers, including NPAR, SIRI and NLR, a trend towards increased mortality risk was observed in the minimally adjusted models (Model 1 and 2). However, these associations were substantially attenuated and lost statistical significance after full adjustment for covariates in Model 3 (All *P* for trend > 0.05). MLR did not show a significant association with all-cause mortality in any of the models when analyzed as a categorical variable. Sensitivity analyses using different variable transformations (continuous vs. categorical) yielded consistent findings regarding the prognostic value of ALI, NPAR, SIRI and MLR. For adjusted model 3, we used RCS COX models to demonstrate the associations between inflammation indices and the risk of all-cause mortality. The results indicated that these markers exhibited a nonlinear association with the risk of BC mortality (**Figure 5**).

**Table 4:** Association of the four inflammatory markers and breast cancer all-cause mortality, among US breast cancer participants, NHANES, 2005 to 2018.

Variables	All-cause death		Breast cancer HR (95%CI, p-value)		
			Model 1	Model 2	Model 3
ALI					
Per 1-unit			0.98(0.97, 0.99) ***	0.98 (0.97, 0.99) ***	0.99 (0.98, 1.00), 0.01
T1		76/220(34.55)	Reference	Reference	Reference
T2		32/147(21.77)	0.57 (0.34, 0.96), 0.03	0.73 (0.44, 1.21), 0.22	0.74 (0.44, 1.24), 0.25
T3		15/118(12.71)	0.31(0.15, 0.65), 0.002	0.32 (0.16, 0.64),0.001	0.41 (0.18, 0.90), 0.03
P for trend		<0.001	<0.001	0.001	0.02
NPAR					
Per 1-unit			1.17(1.07, 1.27) ***	1.15 (1.06, 1.25) ***	1.11 (0.99, 1.23), 0.07
T1		21/117(17.95)	Reference	Reference	Reference
T2		36/159(22.64)	1.47 (0.75, 2.88), 0.26	1.45 (0.77, 2.79), 0.26	1.05 (0.52, 2.09), 0.89
T3		66/209(31.58)	2.16 (1.09, 4.29),0.02	1.99 (1.07, 3.69),0.02	1.25 (0.61, 2.54), 0.54
P for trend		<0.001	<0.001	0.01	0.64
SIRI					
Per 1-unit			1.38 (1.22, 1.57), ***	1.31(1.16, 1.50), ***	1.19 (0.98, 1.45),0.07
T1		18/105(17.14)	Reference	Reference	Reference
T2		34/155(21.94)	1.26 (0,69, 2.33), 0.44	1.16 (0.62,2.17),0.64	0.91 (0.43, 1.95), 0.82
T3		71/225(31.55)	2.60(1.47, 4.58), 0.001	2.10(1.20, 3.66), 0.009	1.50 (0.75, 2.99), 0.24
P for trend		0.004	<0.001	0.002	0.08
AISI					
1-unit			1.00 (1.00, 1.00) ***	1.00 (1.00, 1.00) ***	1.00 (1.00, 1.00), 0.05
100-unit			1.10 (1.05, 1.14) ***	1.09 (1.05, 1.12) ***	1.08 (1.00, 1.17), 0.05
T1		23/129(17.82)	Reference	Reference	Reference
T2		37/166(22.29)	1.21 (0.67, 2.20), 0.52	1.08 (0.60, 1.95), 0.79	0.78 (0.40, 1.53),0.48
T3		63/190(33.16)	2.10 (1.20, 3.67), 0.009	1.70 (1.00, 2.93),0.05	1.37 (0.72, 2.59), 0.32
P for trend		0.005	0.006	0.03	0.16
NLR					
1-unit			1.21 (1.10, 1.33) ***	1.20 (1.10, 1.31)***	1.09 (0.95, 1.26), 0.19
T1		17/107(15.89)	Reference	Reference	Reference
T2		33/151(21.85)	1.43 (0.73, 2.82), 0.29	1.34 (0.71, 2.52),0.36	1.20 (0.59, 2.43),0.60
T3		73/227(32.16)	2.29 (1.20, 4.35), 0.01	1.99 (1.07, 3.70),0.02	1.37 (0.70, 2.68), 0.35
P for trend		0.005	0.009	0.02	0.34
MLR					
1-unit			4.24 (2.10, 8.57) ***	4.21(2.17, 8.14) ***	4.43(1.81, 10.82), 0.001
T1		13/76(17.11)	Reference	Reference	Reference
T2		32/150(21.33)	0.98 (0.42, 2.26), 0.95	0.80 (0.33, 1.97), 0.64	0.92 (0.34, 2.47),0.87
T3		78/259(30.11)	1.48 (0.72, 3.04), 0.28	1.36 (0.64, 2.93),0.42	1.40 (0.57, 3.42), 0.45
P for trend		0.01	0.10	0.08	0.17

Model 1 was unadjusted for none. Model 2 was adjusted for age, race, and Model 3 was adjusted for age, race, education, marital status, PIR, BMI and diabetes. Model 3 * (for ALI) was adjusted for age, race, education, marital status, PIR and diabetes (Because the definition of ALI includes BMI). ***p < 0.001.

### 3.4 Comparison of predictive performance of the inflammation markers for BC risk and all-cause mortality

**Figure 6:**
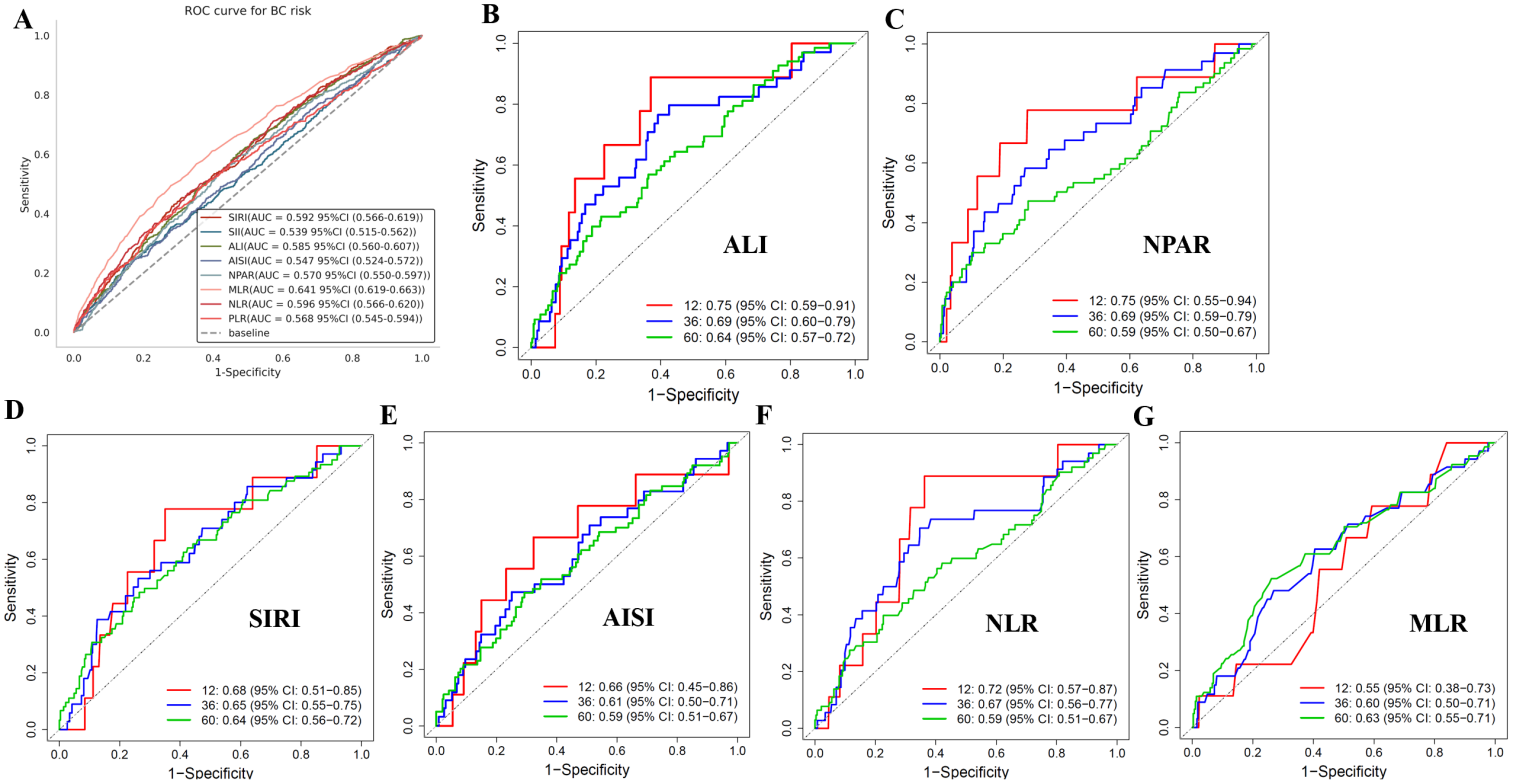
Comparison of predictive performance of inflammation markers for BC risk, all-cause mortality. **(A)** Receiver operating characteristic curve (ROC) analyses for breast cancer patient. **(B–G)** Time-dependent ROC curve of ALI/NPAR/SIRI/AISI/NLR/MLR for all-cause mortality.

ROC curves were employed to compare the predictive performance of inflammation markers in BC patients. The AUC values for these indicators were as follows (**Figure 6A**): SIRI AUC = 0.592 (95%CI 0.566-0.617), ALI AUC = 0.585 (95%CI 0.560-0.611), NPAR AUC = 0.570 (95%CI 0.550-0.597), AISI AUC = 0.547 (95%CI 0.521-0.573), MLR AUC = 0.641 (95%CI 0.619-0.663), NLR AUC = 0.596 (95%CI 0.566-0.620). Similar to the results of **Tables 2** and **3**, ALI, NPAR, SIRI, MLR, NLR had better prediction ability for breast cancer risk. Based on time-dependent ROC curves, the results demonstrated that ALI and NPAR displayed the best prediction performance for BC all-cause mortality, followed by SIRI and NLR with good predictive ability as well (**Figures 6B–G**). As shown in **Figure 6B**, the AUC for ALI predicting all-cause mortality was 0.69 at 3-year survival during follow-up period (FPS), and NPAR AUC = 0.69, NLR AUC = 0.67.

### 3.5 The construction of breast cancer risk prediction ML models based inflammatory status

**Figure 7:**
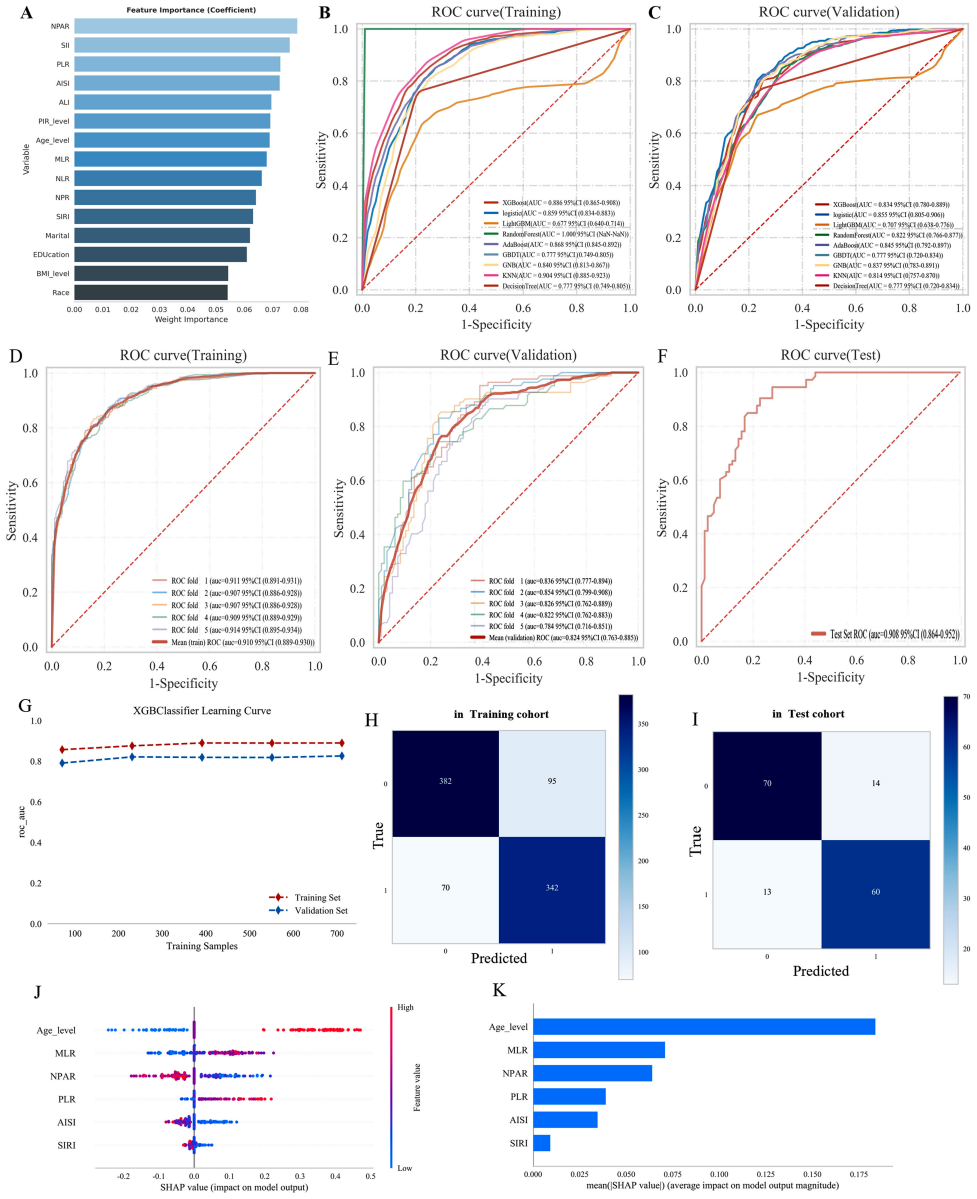
Predictive performance of inflammatory markers for breast cancer and the construction of machine learning (ML) algorithm models for breast cancer patient. **(A)** Importance matrix and SHAP summary plot displayed the contributions of all variables to the XGBoost model. **(B, C)** Clarification of machine learning models; **(D-F)** ROC curves of XGBoost model in the training, validation and test set; **(G)** Learning curve of model building process; **(H, I)** Confusion matrix of XGBoost model in the training and test set. **(J, K)** SHAP summary plot displayed the contributions of XGBoost model feature variables. Group, 0, control group; 1, breast cancer.

The relevance of the variables included was predicted through the construction of a machine learning model using the XGBoost method, and NPAR was shown to be the most significant factor on the prevalence of BC (**Figure 7A**). Furthermore, our study established the optimal machine learning model based on each variable. By comparative evaluation of multiple ML models in classification modeling using clinical parameters (**Figures 7B, C**; **Supplementary Table 3**), including XGBoost, Logistic regression, gradient boosting, random forest and so on, and further excluding models with overfitting risks, we found that the XGBoost models performed stably in both the test and validation sets (**Figures 7D–F**). Based on NPAR, SIRI, AISI, MLR, PLR and Age_level, the model’s efficacy was assessed through 5-fold cross-validation on the training data, yielding an AUC of 0.910 (95%CI: 0.889-0.930). Our study further generated a simple risk prediction model online webpage (https://www.xsmartanalysis.com/model/list/predict/model/html?mid=29378&symbol=9Hr1762Lz26dA628qK00, **Supplementary Figure 3**). Upon applying the final model to the test set, the AUC achieved was 0.908 (95%CI: 0.864-0.952) with an accuracy of 0.828, and F1 score of 0.816.

To address the potential dependence of model performance on a single train-test split, we additionally performed nested cross-validation, which yielded a consistent mean AUC of 0.852 (SD = 0.025; **Supplementary Table 4**), further supporting the model’s generalizability.

### 3.6 The validation of the breast cancer risk prediction XGBoost model

**Figure 8:**
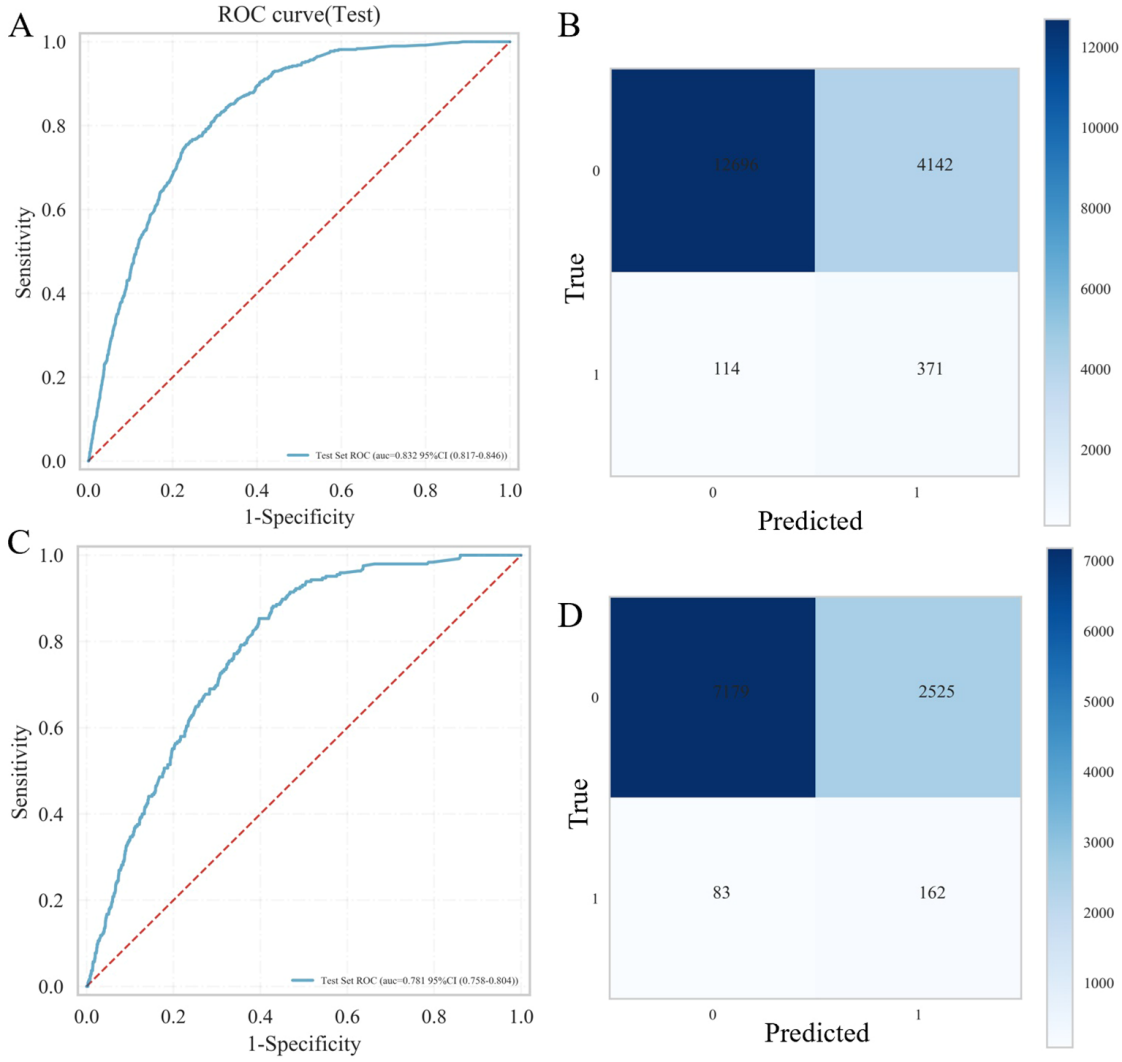
The validation of the XGBoost model for breast cancer patient. **(A)** ROC curves of XGBoost model in the validation set; **(B)** Confusion matrix of XGBoost model in the validation set. **(C, D)** ROC curves and Confusion matrix of XGBoost model in the external validation set: other periods in the NHANES database.

To further evaluate the discrimination ability of the developed model in the entire NHANES population, the trained XGBoost model was subsequently applied to all eligible NHANES participants (2005–2018), yielding an AUC of 0.832 (95% CI: 0.817–0.846, **Figures 8A, B**). Furthermore, two different external validation strategies were used in this study, including independent NHANES survey cycles and a Chinese cohor. We utilized other periods in the NHANES database (1999-2000, 2001-2002, 2003-2004 and 2017-March 2020 Pre-Pandemic), including 9704 female controls and 245 as an external validation set to analyze the prediction ability of XGBoost model. Results demonstrated that XGBoost model had good and stable risk predictive performance for BC, displaying the AUC of 0.781 (95%CI: 0.758-0.804) and accuracy of 0.738 in validation set (**Figures 8C, D**).

### 3.7 The cross-ethnic external validation of the breast cancer risk prediction XGBoost model

**Figure 9:**
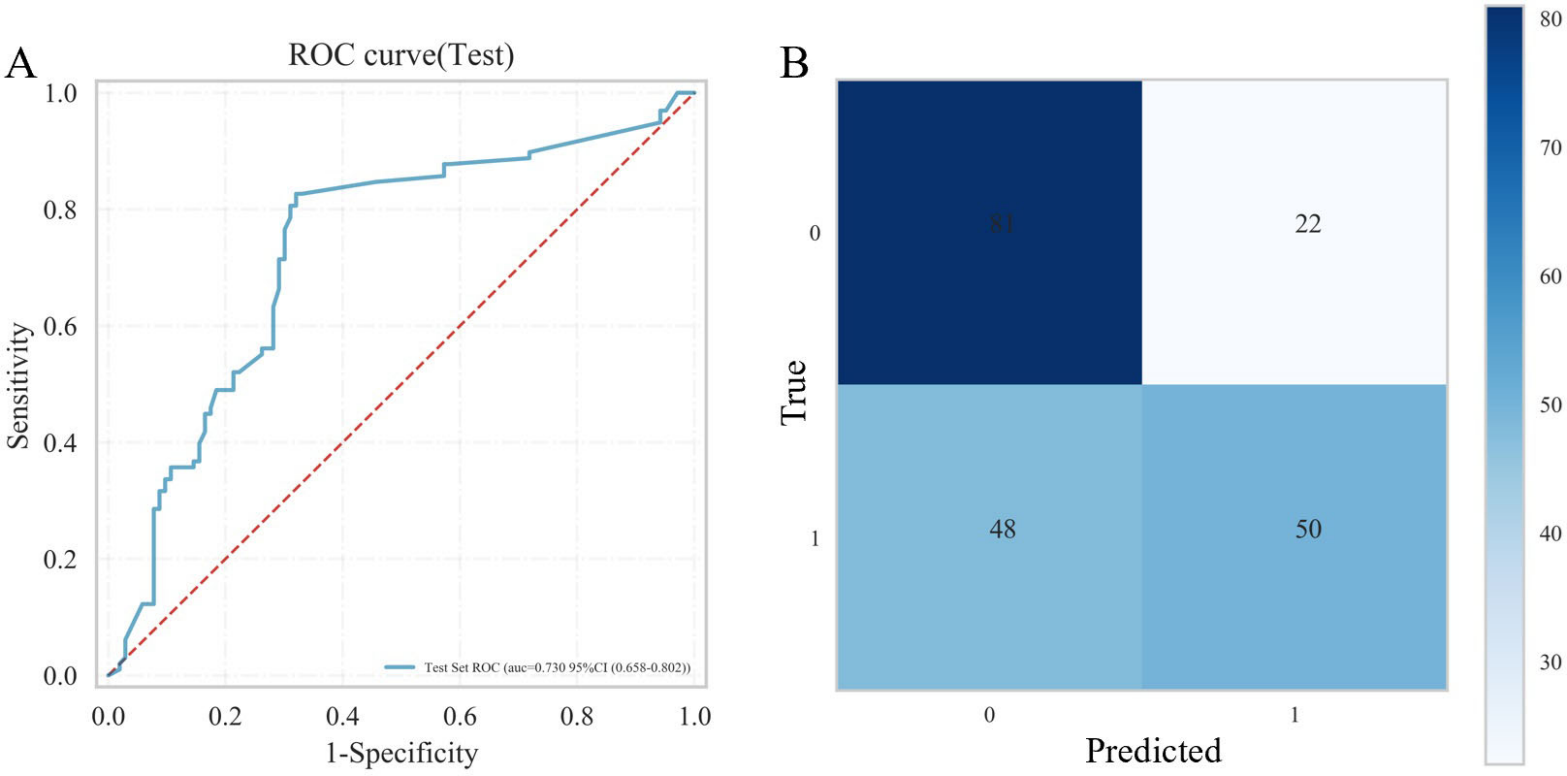
The cross-ethnic external validation of the XGBoost model for breast cancer patient. **(A)** ROC curves of XGBoost model in the external validation set; **(B)** Confusion matrix of XGBoost model in the external validation set.

Furthermore, we performed the external cohort validation recruited from The Second Affiliated Hospital of Xi’an Jiaotong University, including 98 cases of breast cancer and 103 control participants. Results demonstrated this XGBoost model showed a certain level of predictive ability, with the AUC of 0.730 (95%CI: 0.658-0.802) and accuracy of 0.661 (**Figures 9A, B**).

## 4 Discussion

Inflammation has emerged as a pivotal player in the intricate interplay between cancer initiation, progression, and prognosis. This study, based on the large-scale, nationally representative data from NHANES, provided a comprehensive evaluation of the prognostic value of many inflammatory indices in breast cancer. The NHANES, a continuously conducted survey of a representative sample of the non-institutionalized civilian US population, provides a wealth of data on health and nutritional status, including inflammation markers. Recent NHANES-based studies have shed light on the associations between inflammation status markers and cancer incidence and survival in the general population, as well as in adult cancer survivors. One such study utilized NHANES data to investigate the relationship between NLR and cancer incidence (19). The results showed that individuals with elevated NLR had a significantly higher risk of developing various cancers, including breast cancer, compared to those with normal NLR (19, 20). This finding supported the notion that systemic inflammation was an early event in carcinogenesis and may serve as a modifiable risk factor.

Our study demonstrated that readily available peripheral blood indices, particularly those integrating nutritional status like the ALI and the novel NPAR, served as robust predictors of both breast cancer risk and all-cause mortality. The findings could confirm and extend the established link between systemic inflammation and cancer outcomes (21). The significant associations observed for SIRI, AISI, and NLR align with a large body of evidence implicating neutrophils and platelets in tumor progression through the promotion of angiogenesis, metastasis, and immune suppression. Neutrophils promoted metastasis via matrix metalloproteinase secretion (22, 23). Studies indicated that platelets released diverse molecules from platelets and further contributed to an inflammatory state by activating various normal cells, such as fibroblasts, immune cells, and vascular cells, and could facilitate circulating tumor cell adhesion (24). At present, exploring the role of the tumor microenvironment (TME) and peripheral blood immune status composed of immune cells in tumor progression and risk prognosis prediction would have significant importance (25, 26).

Conversely, the protective association of ALI underscores the critical interplay between nutrition and inflammation, suggesting that nutritional status, reflected by albumin and BMI, may modulate the host’s inflammatory response and its impact on survival (27) (28). A novel contribution of our study is the identification of NPAR as a significant prognostic factor. As a composite marker encompassing myeloid inflammation (neutrophils, platelets) and nutritional status (albumin), NPAR may offer a more holistic reflection of the pro-tumor systemic environment than ratios based solely on cellular components. This empirically validates the “nutrition-inflammation axis” in BC progression, suggesting albumin supplementation could partially mitigate inflammation-mediated risk, potentially through impacts on immunity, oxidative stress, and drug pharmacokinetics. Hypoalbuminemia acted as a key pathway linking systemic inflammation to poor outcomes (29, 30). Interventions aimed at maintaining or improving albumin levels warrant investigation. Serum albumin demonstrated significant anti-inflammatory and prognostic value in cancer through multiple interconnected mechanisms. Albumin modulated immune responses by binding damage-associated molecular patterns, reducing NF-κB activation and pro-inflammatory cytokine secretion (31). In the TME, albumin-based nanoparticles could be engineered to reprogram immunosuppressive macrophage. Manganese-bound albumin activates the TLR4-TRIF pathway, inducing pro-inflammatory polarization of tumor-associated macrophages and enhancing CD8+ T-cell infiltration in non-small cell lung cancer (32). Furthermore, albumin serves as a critical pharmacokinetic regulator for monoclonal antibodies, as its half-life correlates with IgG catabolism; higher baseline albumin levels predict improved response to immune checkpoint blockade across cancer types, likely by reducing antibody clearance (33). These mechanisms underscore albumin’s dual role as a biomarker and therapeutic vehicle in inflammation-driven cancer progression (34). Future studies should validate thresholds in prospective cohorts and explore stage-specific effects. For instance, tracking ALI/NPAR during chemotherapy to preempt cachexia-inflammation imbalance, or testing albumin supplementation or anti-inflammatories (e.g., IL-6 inhibitors) in high-SIRI/AISI patients.

These inflammation markers including ALI, NPAR, SIRI, AISI, NLR and MLR could act as the potential risk and prognostic markers of breast cancer, suggesting the underlying mechanisms associated with the dynamic tumor microenvironment. Neutrophils can be polarized to a pro-tumor phenotype, facilitating metastasis through the release of extracellular traps and matrix-degrading enzymes (35). Monocytes were recruited to tumors and partially differentiated into macrophages, and underwent transcriptional reprogramming resulting in tumor-promoting functions, including suppression of anti-tumor T-cell responses, promotion of angiogenesis, and facilitation of metastatic colonization (36). Mate et al. reported cancer-associated remodeling of the epigenomic landscape in peripheral monocytes could influence the gene expression programs they acquired in the tumor (37). Lymphocytes, as the cornerstone of anti-tumor immunity, were reflected in ratios like MLR and NLR; their reduction signifies impaired immune surveillance, allowing for tumor escape (38).

A paramount finding of our study was the development and multi-validation of an XGBoost machine learning model. The model exhibited robust and good predictive performance in NHANES data from other time periods, demonstrating its reliability in the source population of this study. However, when we applied this model to an independent Chinese cohort consisting strictly of untreated newly diagnosed breast cancer patients and healthy controls, we observed that although the model maintained an acceptable overall differentiation ability (AUC = 0.730), its sensitivity and accuracy in identifying BC patients were significantly insufficient. This result was due to the significant difference between the baseline levels of inflammatory indicators in the Chinese and American cohorts. The inflammatory levels in the Chinese health and BC cohorts were generally lower than those in the NHANES data, which was the key to the failure of the model decision threshold in the Chinese population. Therefore, the suboptimal sensitivity revealed a fundamental challenge: biomarkers and the models built upon them were often calibrated to the baseline characteristics of the source population. Differences in genetic background, lifestyle, and average inflammatory tone could lead to critical miscalibration when applied to new groups. These findings suggested the more efforts focused on population-specific model calibration and threshold optimization, particularly for multi-ethnic nations and immigrant-receiving countries in future. In diverse populations, a risk prediction model calibrated on a single ethnic group may perpetuate health disparities by under-serving minority populations. Therefore, our work underscores that for the equitable implementation of precision public health, developing ethnicity-specific calibration, or establishing dynamic, adjustable thresholds based on individual baseline characteristics, is not merely an improvement but a necessity. Meanwhile, these readily available inflammatory indices could serve as a potential foundation for cancer risk prediction models, and further to integrate more specific cancer biomarkers (e.g., cancer-specific antigens, genetic markers) could ensure broad accessibility and better accuracy.

Several limitations in this study must be acknowledged. The lack of detailed cancer stage, treatment history, and cancer-specific mortality data in NHANES prevented more nuanced analysis, and the reliance on participants’ self-reported history of cancer could introduce reporting bias. The sample size for the cross-ethnic validation was relatively small, and the generalizability of our findings to other ethnic groups requires further confirmation. Furthermore, as with any observational study, residual confounding cannot be entirely ruled out. Future multi-center prospective studies with standardized inflammatory biomarker measurements and longitudinal follow-up are needed to further validate the model’s clinical utility and generalizability across diverse populations.

## 5 Conclusion

This study demonstrated the role of many inflammation indices as powerful, non-invasive biomarkers for breast cancer risk stratification and prognosis. The ALI and NPAR indices, in particular, highlighted the prognostic significance of the nutrition-inflammation axis. The validated machine learning model integrating NPAR, SIRI, AISI, MLR, PLR and Age, could provide a practical tool for individualized BC risk prediction, and indicate that inflammatory indicators as core components worthy of inclusion in the cancer prediction framework. Most importantly, our cross-ethnic validation offered a critical roadmap for the future of biomarker development, emphasizing that the cancer risk model must be paved with careful calibration to ensure that precision medicine benefits all populations equitably. Future research would construct more superior cancer prediction models, and explore the biological mechanisms linking these specific inflammatory indices and albumin to BC progression and comorbidity development, and assess the cost-effectiveness of implementing these markers and the risk model in routine clinical practice for BC survivorship care.

## Data Availability

The original contributions presented in the study are included in the article/**Supplementary Material**. Further inquiries can be directed to the corresponding author.
