# Supplementary material for: From associations to clinical practice: translating inflammatory-nutritional indices into a machine learning-driven model for breast cancer risk stratification with cross-ethnic validation
Source: Front Immunol. 2026 Jul 15;17:1845883. doi: 10.3389/fimmu.2026.1845883 (PMC13416066; doi:10.3389/fimmu.2026.1845883)
Supplement: Supplementary file 1 [file DataSheet1.docx]

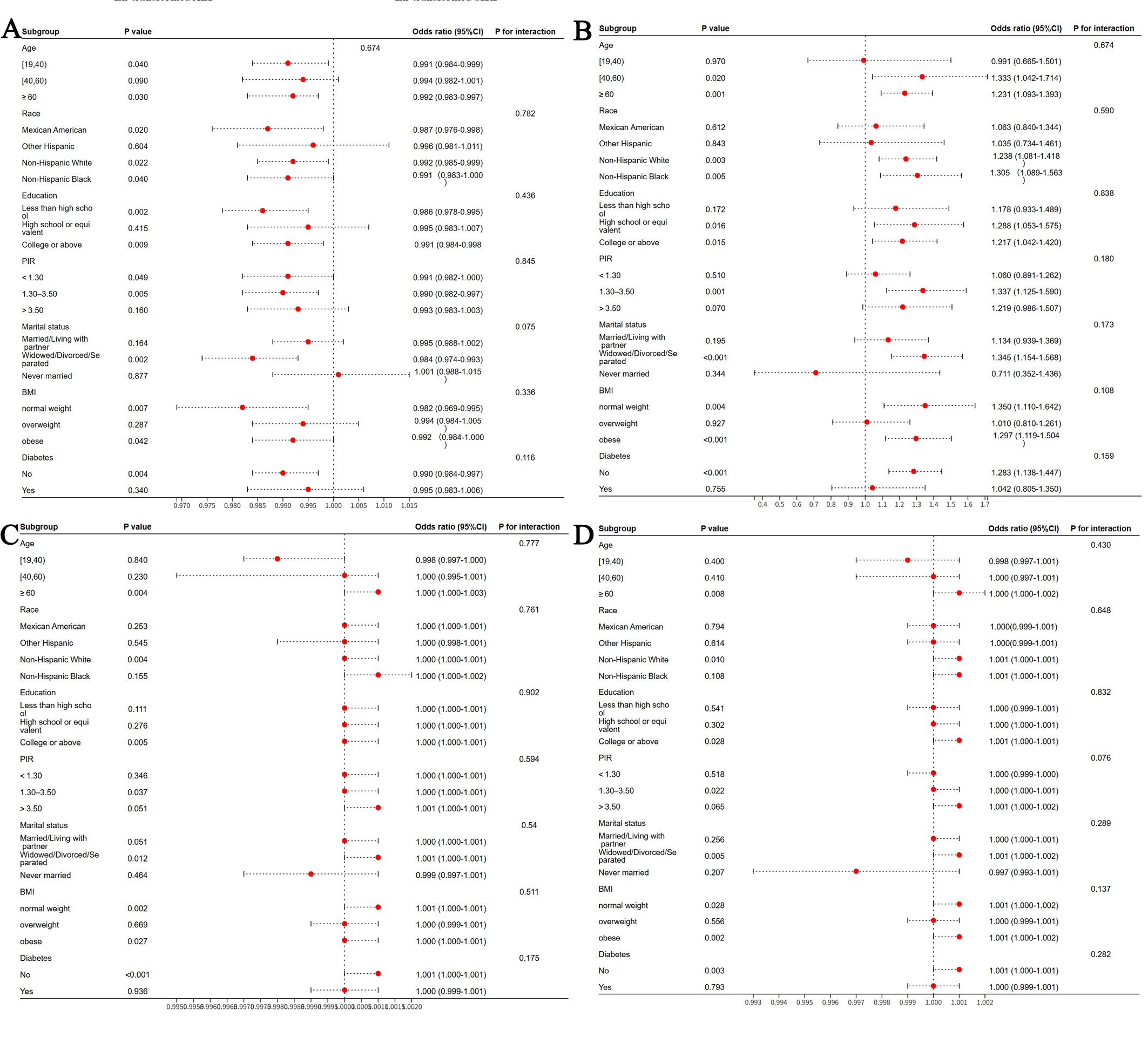


**Figure S1. Forest plot for stratified associations analysis between ALI (A), SIRI, SII (C) and AISI (D) and breast cancer all-cause mortality.**

All covariates (consistent with Model 3) were adjusted in this model without adjusting the stratified variable itself. No significant interactions were found between those inflammatory markers and these stratification variables.


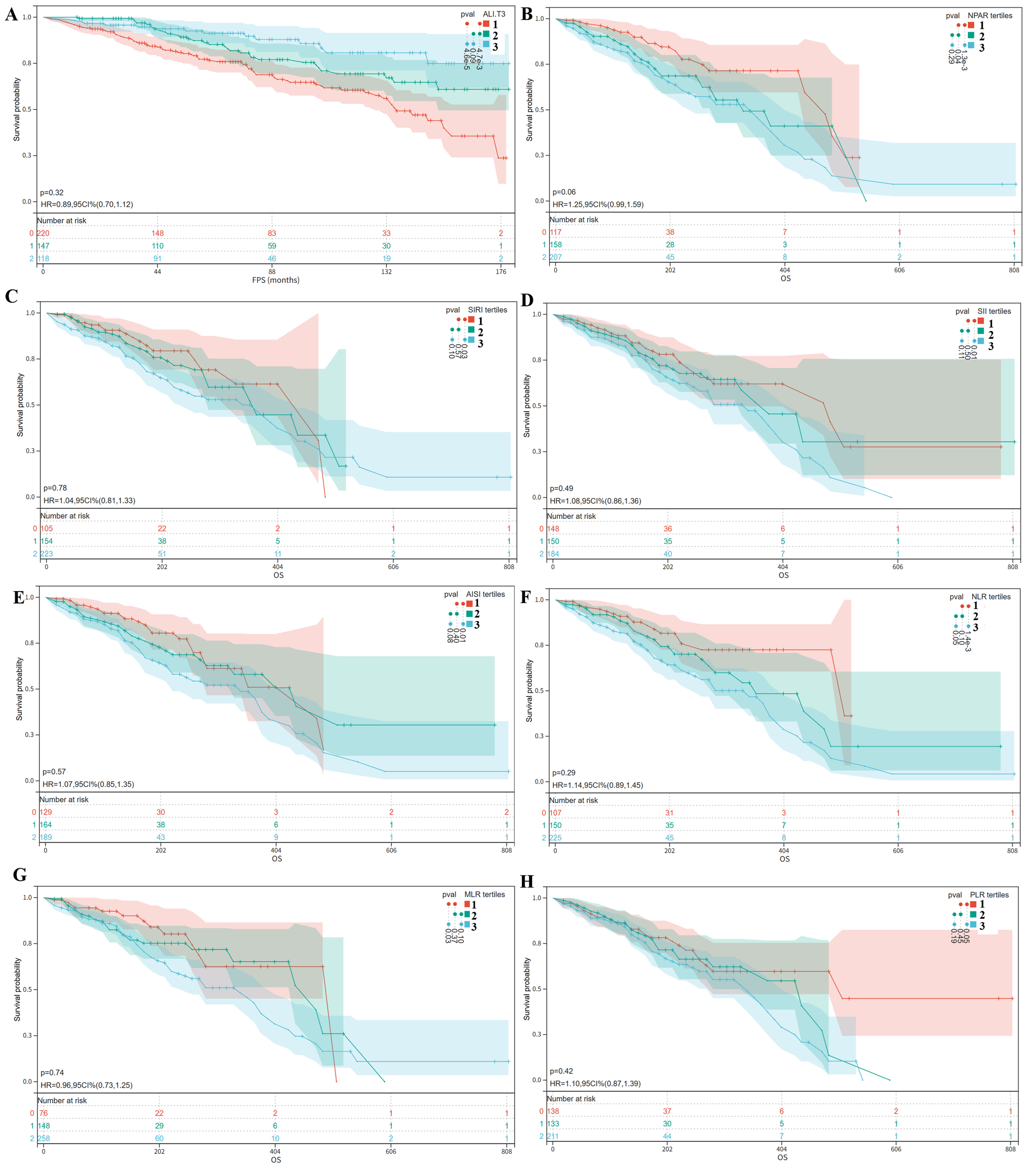


**Figure S2. Association of the inflammation markers with breast cancer all-cause mortality based on the Kaplan-Meier survival analysis.**

(A-H) Kaplan-Meier survival curves for all-cause mortality overall survival (OS) stratified by ALI/NPAR/SIRI/SII/AISI/NLR/MLR/PLR tertiles. HR, hazard ratio.


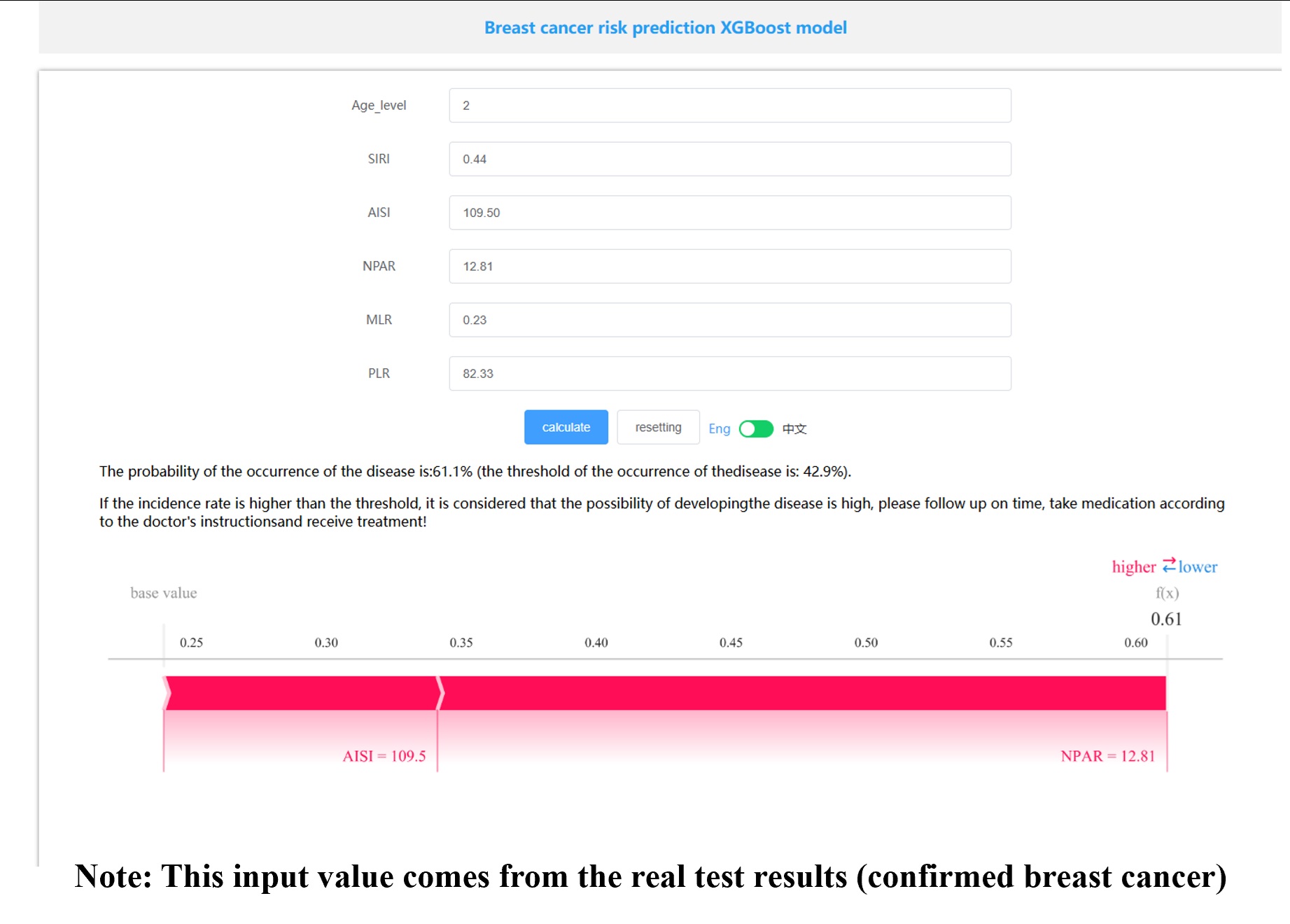


**Figure S3. The simple breast cancer risk prediction XGBoost model online webpage.** (https://www.xsmartanalysis.com/model/list/predict/model/html?mid=29378&symbol=9Hr1762Lz26dA628qK00). This input value comes from the real test results (confirmed breast cancer).
