# Supplementary material for: From associations to clinical practice: translating inflammatory-nutritional indices into a machine learning-driven model for breast cancer risk stratification with cross-ethnic validation
Source: Front Immunol. 2026 Jul 15;17:1845883. doi: 10.3389/fimmu.2026.1845883 (PMC13416066; doi:10.3389/fimmu.2026.1845883)
Supplement: Supplementary file 2 [file Table1.docx]

**Table S1. Baseline characteristics of participants by cancer status.**

| **Variables** | **Overall (*n* = 40926)** | **Non-cancer (*n* = 37144)** | **Cancer (*n* = 3782)** | ***p*-value** |
| --- | --- | --- | --- | --- |
| **Age (mean (SD))** | 48.83 (18.48) | 47.10 (17.98) | 65.79 (14.18) | <0.001 |
| **Age_level (%)** |  |  |  | <0.001 |
| [19,40**)** | 14644 (35.8) | 14392 (38.7) | 252 (6.7) |  |
| [40,60**)** | 12802 (31.3) | 12021 (32.4) | 781 (20.7) |  |
| ≥ 60 | 13480 (32.9) | 10731 (28.9) | 2749 (72.7) |  |
| **Gender (%)** |  |  |  | 0.050 |
| Male | 19858 (48.5) | 18082 (48.7) | 1776 (47.0) |  |
| Female | 21068 (51.5) | 19062 (51.3) | 2006 (53.0) |  |
| **Race**, **(%)** |  |  |  | <0.001 |
| Mexican American | 6514 (15.9) | 6257 (16.8) | 257 (6.8) |  |
| Other Hispanic | 3945 (9.6) | 3714 (10.0) | 231 (6.1) |  |
| Non-Hispanic White | 16843 (41.2) | 14315 (38.5) | 2528 (66.8) |  |
| Non-Hispanic Black | 8929 (21.8) | 8372 (22.5) | 557 (14.7) |  |
| Other Race | 4695 (11.5) | 4486 (12.1) | 209 (5.5) |  |
| **BMI** **g/m^2^,mean(SD)** | 29.08 (6.99) | 29.08 (7.04) | 29.06 (6.55) | 0.832 |
| **Education**, **n(%)** |  |  |  | <0.001 |
| Less than high school | 10090 (25.4) | 9251 (25.8) | 839 (22.2) |  |
| High school or equivalent | 9102 (22.9) | 8264 (23.0) | 838 (22.2) |  |
| College or above | 20496 (51.7) | 18396 (51.2) | 2100 (55.6) |  |
| **PIR**, **n(%)** |  |  |  | <0.001 |
| < 1.30 | 12016 (29.4) | 11137 (30.0) | 879 (23.2) |  |
| 1.30–3.50 | 13973 (34.1) | 12591 (33.9) | 1382 (36.5) |  |
| > 3.50 | 14937 (36.5) | 13416 (36.1) | 1521 (40.2) |  |
| **Marital status**, **n(%)** |  |  |  | <0.001 |
| Married/Living with partner | 23512 (58.7) | 21292 (58.8) | 2220 (58.7) |  |
| Widowed/Divorced/Separated | 9006 (22.5) | 7693 (21.2) | 1312 (34.7) |  |
| Never married | 7468 (18.7) | 7223 (19.9) | 244 (6.5) |  |
| Other | 34 (0.1) | 28 (0.1) | 6 (0.1) |  |
| **Diabetes**, **n (%)** |  |  |  | <0.001 |
| Yes | 5147 (12.9) | 4375 (12.0) | 772 (21.2) |  |
| No | 34844 (87.1) | 31979 (88.0) | 2865 (78.8) |  |
| **ALI (mean (95%CI** **of mean))** | 70.58 (69.33, 71.84) | 71.24 (70.08, 72.40) | 66.21 (62.35, 69.65) | 0.01 |
| **SIRI (mean (95%CI of mean))** | 1.223 (1.213, 1.233) | 1.221 (1.212, 1.231) | 1.466 (1.424, 1.507) | <0.001 |
| **SII (mean (95%CI of mean))** | 537.6 (533.5, 541.7) | 536.2 (532.3, 540.1) | 594.8 (579.2, 610.3) | <0.001 |
| **AISI (mean (95%CI of mean))** | 313.4 (310.4, 316.3) | 308.8 (305.7, 311.9) | 358.9 (322.5, 374.2) | <0.001 |
| **MLR (mean (95%CI of mean))** | 0.2785 (0.2771, 0.2798) | 0.2756 (0.2743, 0.2769) | 0.3351 (0.3283, 0.3418) | <0.001 |
| **NLR (mean (95%CI of mean))** | 2.149 (2.137, 2.162) | 2.146 (2.134, 2.159) | 2.501 (2.444, 2.557) | <0.001 |
| **PLR (mean (95%CI of mean))** | 126.3 (125.7, 126.8) | 124.8 (124.3, 125.3) | 138.7 (130.6, 141.1) | <0.001 |
| **NPR (mean (SD))** | 0.018 (0.015) | 0.018 (0.015) | 0.019 (0.009) | 0.002 |

**Table S2**. Threshold effect analysis of ALI, SII, SIRI and AISI on breast cancer risk.

| Exposure | OR (95%CI), *p* value | | | |
| --- | --- | --- | --- | --- |
|  | ALI | SIRI | SII | AISI |
| Fitting by weighted regression adjusted model | 0.992 (0.988, 0.995) <0.0001 | 1.210 (1.098, 1.334) 0.0001 | 1.000 (1.000, 1.001) 0.0012 | 1.000 (1.000, 1.001) 0.0370 |
| Fitting by two-piecewise regression adjusted model |  |  |  |  |
| Inflection points (K) | 26.442 | 1.461 | 1149.067 | 607.5 |
| < K ^1^ | 0.934 (0.898, 0.972) 0.0008 | 1.833 (1.350, 2.488) 0.0001 | 1.001 (1.000, 1.001) 0.0002 | 1.001 (1.000, 1.002) 0.0008 |
| > K ^2^ | 0.993 (0.990, 0.997) 0.0003 | 1.054 (0.906, 1.227) 0.4939 | 1.000 (0.999, 1.000) 0.7186 | 1.000 (0.999, 1.000) 0.2793 |
| *p* for likelihood ratio test | 0.006 | 0.005 | 0.028 | 0.007 |

Model was adjusted for age, race, education, marital status, PIR, BMI and diabetes.

Table S3. Performance Comparison of Multiple Models based on Training Set Results.

| Models | AUC(95%CI) | Accuracy(95%CI) | Sensitivity (95%CI) | Specificity (95%CI) | Positive predictive value (95%CI) | Negative predictive value (95%CI) | F1 score (95%CI) |
| --- | --- | --- | --- | --- | --- | --- | --- |
| XGBoost | 0.886 (0.865-0.908) | 0.804(0.799-0.810) | 0.825(0.800-0.850) | 0.787(0.757-0.816) | 0.771(0.751-0.791) | 0.84(0.826-0.854) | 0.796(0.793-0.800) |
| logistic | 0.859 (0.834-0.883) | 0.79(0.781-0.798) | 0.83(0.823-0.838) | 0.755(0.737-0.773) | 0.746(0.733-0.759) | 0.837(0.832-0.843) | 0.786(0.779-0.792) |
| LightGBM | 0.677 (0.640-0.714) | 0.723(0.664-0.782) | 0.652(0.468-0.835) | 0.785(0.696-0.874) | 0.73(0.664-0.795) | 0.744(0.644-0.843) | 0.673(0.569-0.777) |
| RandomForest | 1.000 (NaN-NaN) | 1.0(1.000-1.000) | 1.0(1.000-1.000) | 1.0(1.000-1.000) | 1.0(1.000-1.000) | 1.0(1.000-1.000) | 1.0(1.000-1.000) |
| AdaBoost | 0.868 (0.845-0.892) | 0.79(0.782-0.797) | 0.85(0.832-0.868) | 0.738(0.718-0.757) | 0.737(0.725-0.749) | 0.851(0.838-0.864) | 0.789(0.782-0.797) |
| GBDT | 0.777 (0.749-0.805) | 0.778(0.771-0.786) | 0.759(0.749-0.768) | 0.795(0.787-0.803) | 0.762(0.753-0.770) | 0.792(0.785-0.800) | 0.76(0.752-0.769) |
| GNB | 0.840 (0.813-0.867) | 0.78(0.777-0.783) | 0.809(0.795-0.823) | 0.754(0.745-0.763) | 0.74(0.736-0.744) | 0.821(0.811-0.830) | 0.773(0.768-0.778) |
| KNN | 0.904 (0.885-0.923) | 0.813(0.805-0.822) | 0.854(0.807-0.900) | 0.779(0.730-0.827) | 0.772(0.737-0.808) | 0.863(0.835-0.891) | 0.809(0.800-0.818) |
| DecisionTree | 0.777 (0.749-0.805) | 0.778(0.771-0.786) | 0.759(0.749-0.768) | 0.795(0.787-0.803) | 0.762(0.753-0.770) | 0.792(0.785-0.800) | 0.76(0.752-0.769) |

Table S4. Nested cross-validation results of the XGBoost model.

| Outer_Fold | AUC | Best_nrounds | Best_max_depth | Best_eta |
| --- | --- | --- | --- | --- |
| 1 | 0.8725 | 50 | 3 | 0.1 |
| 2 | 0.8569 | 50 | 3 | 0.1 |
| 3 | 0.8133 | 50 | 3 | 0.1 |
| 4 | 0.8740 | 50 | 3 | 0.1 |
| 5 | 0.8435 | 50 | 3 | 0.1 |
| Mean (SD) | 0.852 (0.025) | | | |
